# Supplementary material for: Exploring the Comprehensive Kozak Sequence Landscape for AAV Production in Sf9 System
Source: Viruses. 2023 Sep 23;15(10):1983. doi: 10.3390/v15101983 (PMC10612025; doi:10.3390/v15101983)
Supplement: Supplementary file 1 [file viruses-15-01983-s001.zip › viruses-2590806-supplementary.pdf]

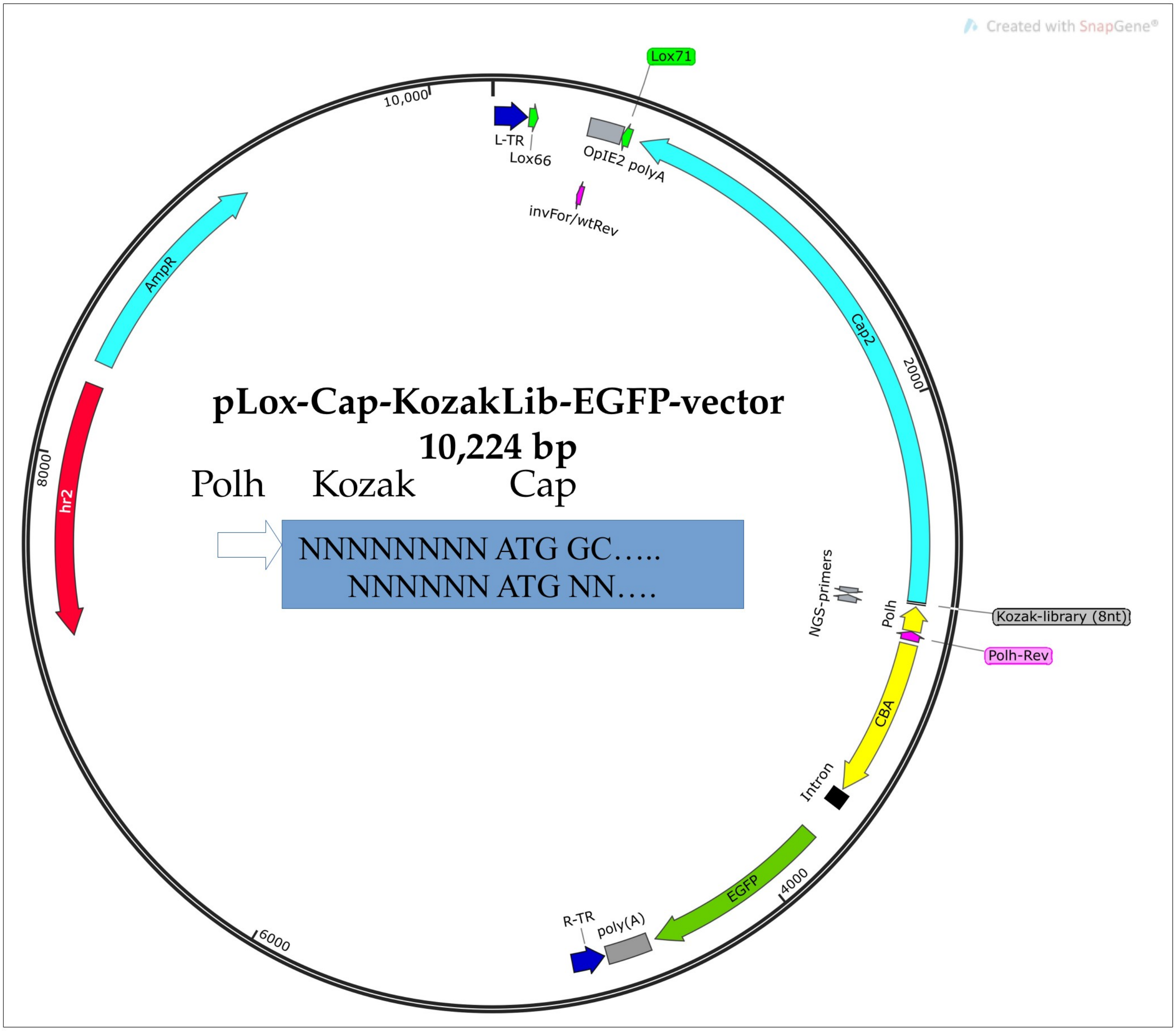


**Figure S1.** pLox-Cap-KozakLib-EGFP vector.

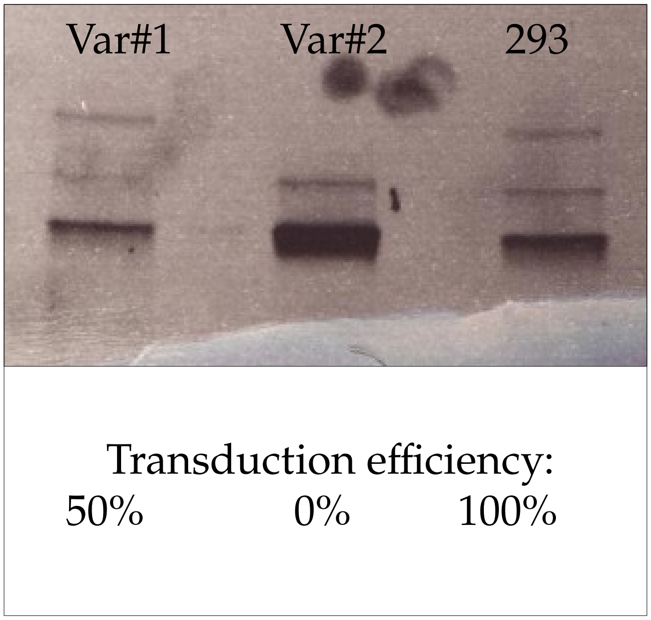


**Figure S2.** VP protein distribution for AAV with Kozak Var#1 and Var#2 produced in Sf9 cells vs AAV2 produced in 293 cells.

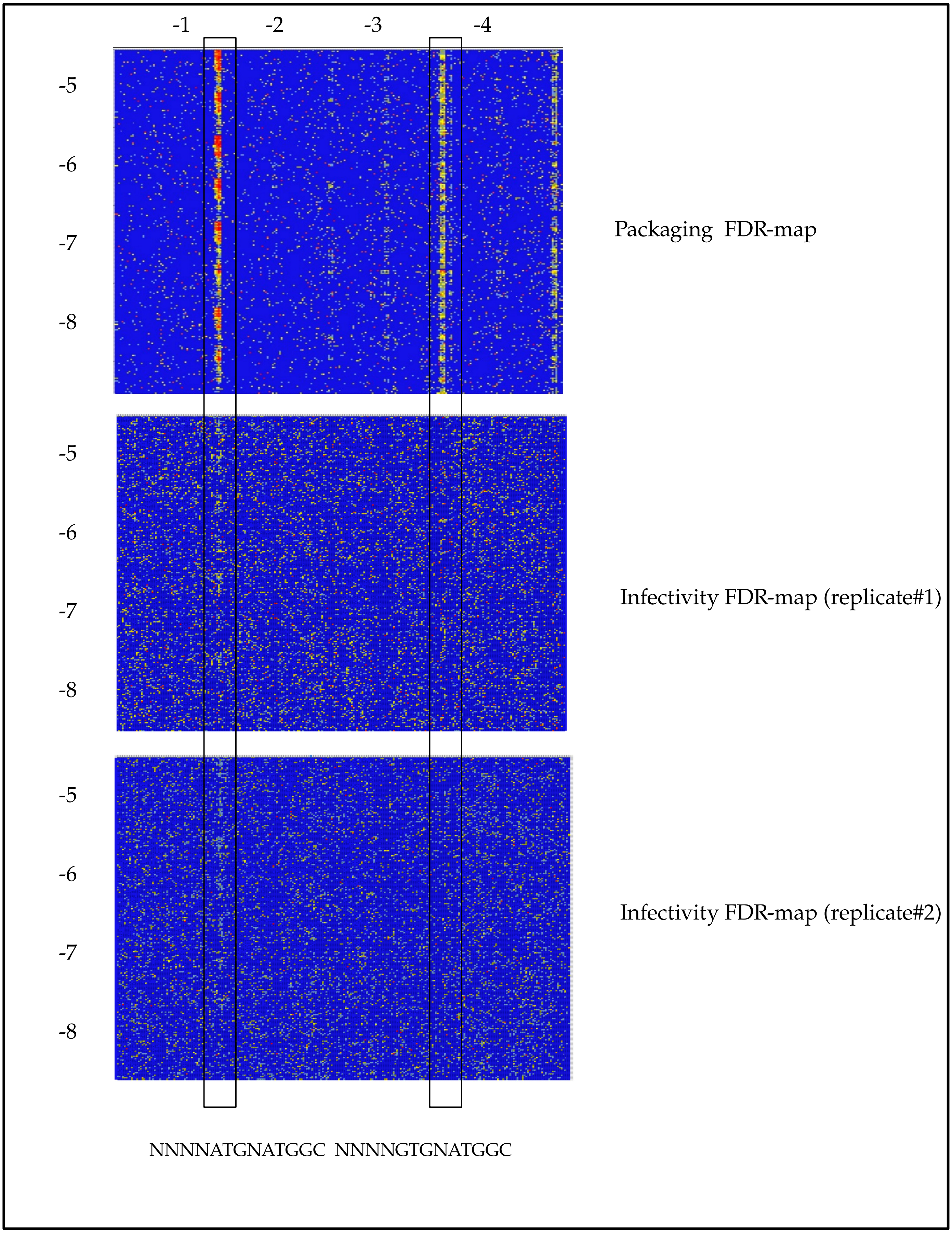


**Figure. S3.** Fractal representation of infectivity of FGDE Kozak library in two biological replicates according to qNGS data.

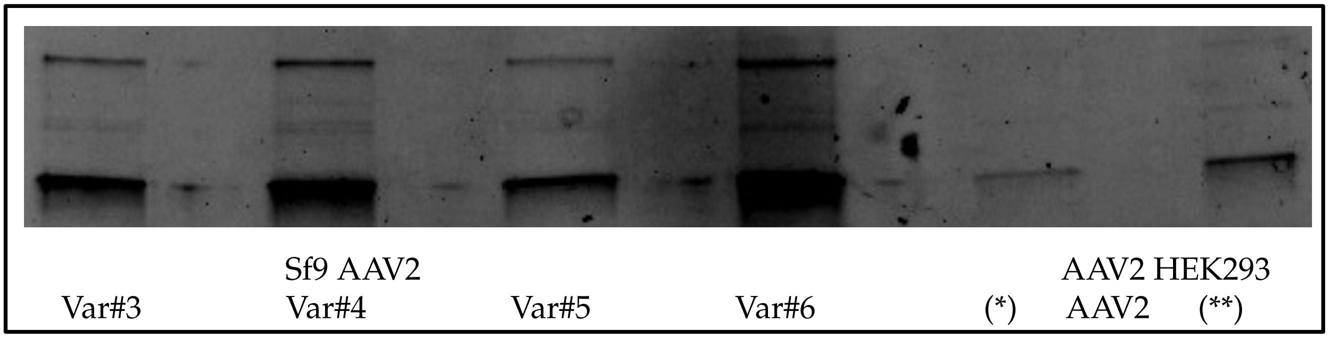


**Figure S4.** PAAG gel of iodixanol purified AAV assembled with the selected Kozak variants (#3-6) into Sf9 cells vs AAV2 assembled into HEK293 cells; * - lower, ** - medium amount of HEK293 AAV2 per line.

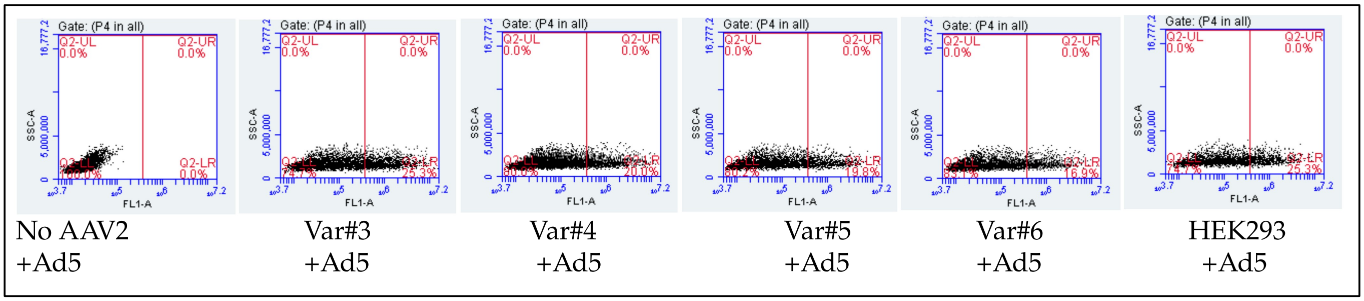


**Figure S5**. Infectivity of AAV packaged in Sf9 cells with selected Kozak variants and in HEK293 cells: C12 cells were transduced by AAV with MOI 10,000 and Ad5 with MOI 5.

**A.**

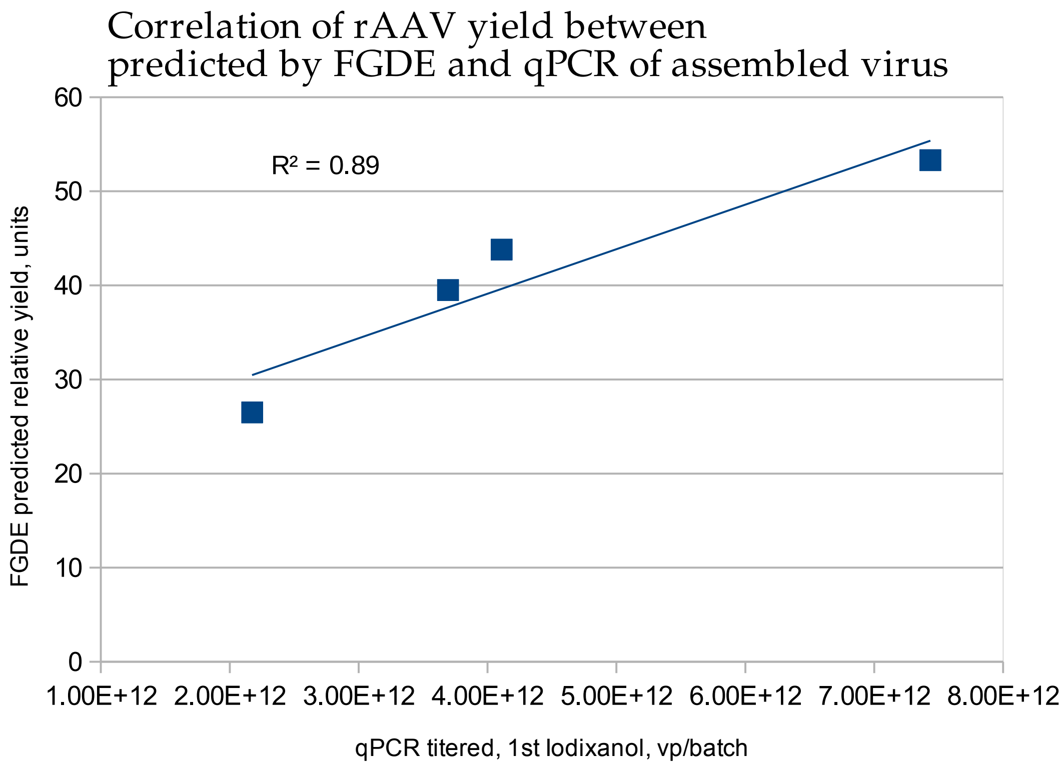


**B.**

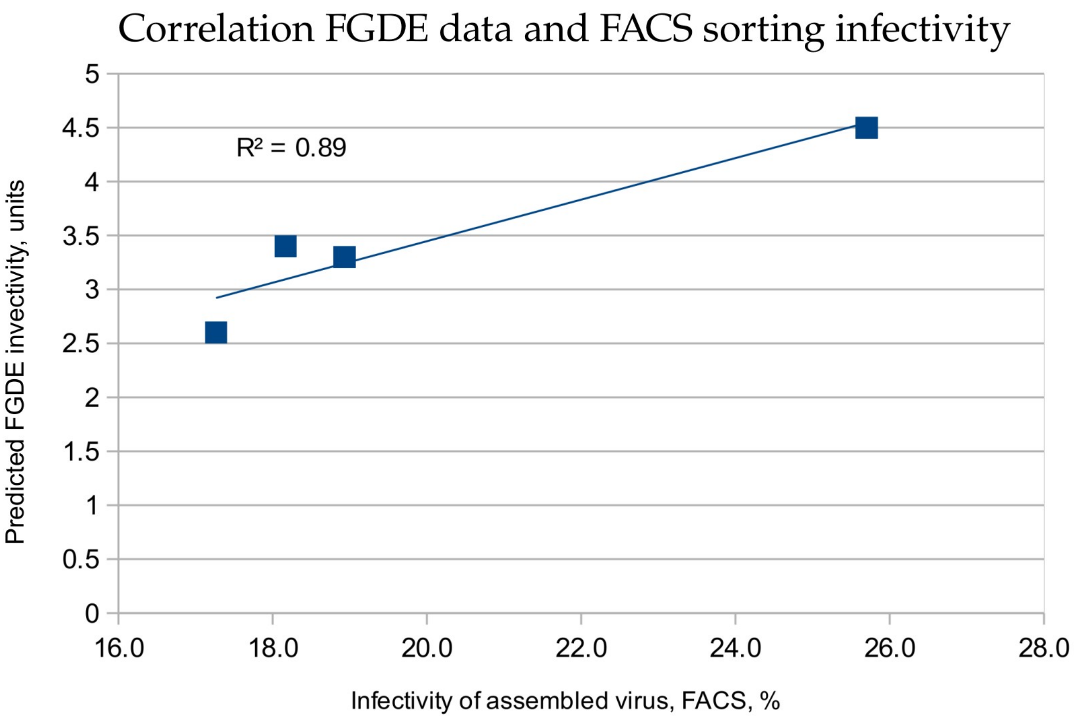


**Figure S6.** Correlation of FGDE approach and routine methods. **A.** Infectivity. **B.** Yield.

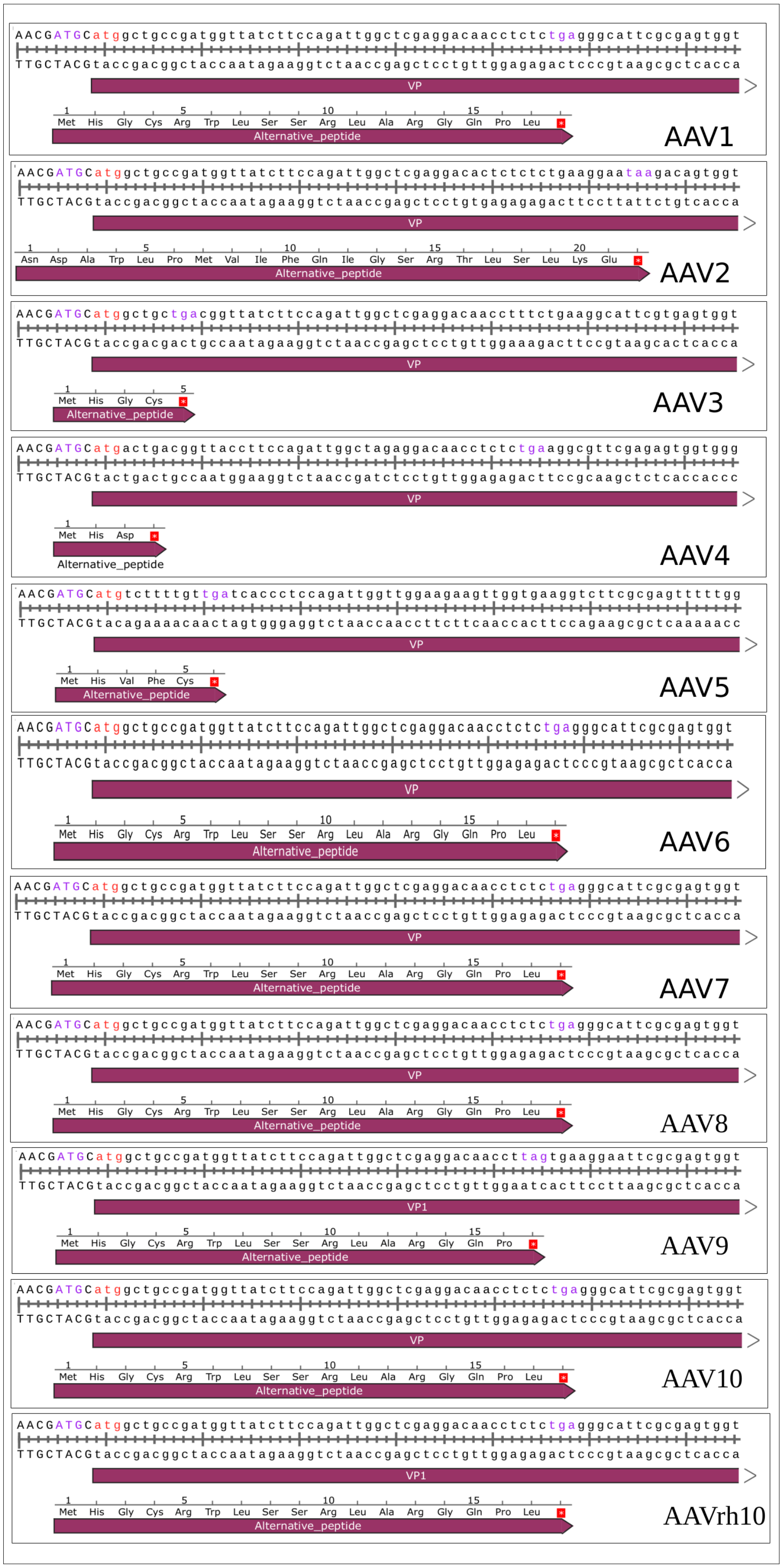


**Figure S7.** Analysis of non-frame up-stream ATG translation in multiple AAV serotypes.

**Table S1.** Pre-selected Kozak varinats based on FGDE approach.

| | Kozak_number | Sequence | seq_pattern | Packaging | Aver_Inf | Log10(Pac) | Log10(Aver) | StDev | 3xiCV | Cluster |
|---|---|---|---|---|---|---|---|---|---|---|
| 1 | 14913 | CAAAATGG | ATGnATG | 108.67 | 1.43 | 2.04 | 0.16 | 0.3 | 1.69 | C1 |
| 2 | 14881 | AGAAATGG | ATGnATG | 93.82 | 1.07 | 1.97 | 0.03 | 0.2 | 2.01 | C1 |
| 3 | 14949 | CGCAATGG | ATGnATG | 93.02 | 2.09 | 1.97 | 0.32 | 0.1 | 10.92 | C1 |
| 4 | 15206 | CGCCATGT | ATGnATG | 83.28 | 1.01 | 1.92 | 0 | 0.2 | 1.4 | C1 |
| 5 | 14885 | AGCAATGG | ATGnATG | 81.49 | 1.07 | 1.91 | 0.03 | 0.1 | 3.86 | C1 |
| 6 | 14886 | AGCCATGG | ATGnATG | 81.19 | 1.31 | 1.91 | 0.12 | 0.4 | 1.14 | C1 |
| 7 | 14914 | CAACATGG | ATGnATG | 80.43 | 1.51 | 1.91 | 0.18 | 0.1 | 7.12 | C1 |
| 8 | 14415 | CATGATGA | ATGnATG | 80.1 | 1.16 | 1.9 | 0.06 | 0.2 | 2.37 | C1 |
| 9 | 15111 | AACGATGT | ATGnATG | 79.42 | 1.79 | 1.9 | 0.25 | 0.4 | 1.48 | C1 |
| 10 | 15169 | CAAAATGT | ATGnATG | 79.28 | 1.95 | 1.9 | 0.29 | 0.3 | 2.13 | C1 |
| 11 | 14919 | CACGATGG | ATGnATG | 76.91 | 2.05 | 1.89 | 0.31 | 0.5 | 1.25 | C1 |
| 12 | 14981 | GACAATGG | ATGnATG | 75.91 | 1.51 | 1.88 | 0.18 | 0.5 | 1.11 | C1 |
| 13 | 14855 | AACGATGG | ATGnATG | 72.35 | 1.34 | 1.86 | 0.13 | 0.1 | 3 | C1 |
| 14 | 14854 | AACCATGG | ATGnATG | 66.78 | 1.85 | 1.82 | 0.27 | 0.1 | 7.91 | C1 |
| 15 | 21307 | ATGGCCAT | other | 64.88 | 1.25 | 1.81 | 0.1 | 0.3 | 1.23 | C1 |
| 16 | 15013 | GGCAATGG | ATGnATG | 64.41 | 2.09 | 1.81 | 0.32 | 0 | 14.04 | C1 |
| 17 | 14863 | AATGATGG | ATGnATG | 64.17 | 1.48 | 1.81 | 0.17 | 0.2 | 2.11 | C1 |
| 18 | 14662 | CACCATGC | ATGnATG | 61.36 | 2.09 | 1.79 | 0.32 | 0.2 | 3.39 | C1 |
| 19 | 15175 | CACGATGT | ATGnATG | 60.66 | 1.65 | 1.78 | 0.22 | 0.5 | 1.14 | C1 |
| 20 | 14982 | GACCATGG | ATGnATG | 58.69 | 1.42 | 1.77 | 0.15 | 0 | 66.7 | C1 |
| 21 | 14657 | CAAAATGC | ATGnATG | 57.21 | 1.46 | 1.76 | 0.16 | 0.4 | 1.16 | C1 |
| 22 | 14862 | AATCATGG | ATGnATG | 55.79 | 1.25 | 1.75 | 0.1 | 0.4 | 1.07 | C1 |
| 23 | 14894 | AGTCATGG | ATGnATG | 55.69 | 1.37 | 1.75 | 0.14 | 0 | 12.87 | C1 |
| 24 | 15045 | TACAATGG | ATGnATG | 55.03 | 1.36 | 1.74 | 0.13 | 0.4 | 1.16 | C1 |
| 25 | 14407 | CACGATGA | ATGnATG | 54.67 | 1.09 | 1.74 | 0.04 | 0.2 | 1.71 | C1 |
| 26 | 15047 | TACGATGG | ATGnATG | 54.4 | 1.65 | 1.74 | 0.22 | 0.5 | 1.03 | C1 |
| 27 | 14951 | CGCGATGG | ATGnATG | 53.34 | 2.62 | 1.73 | 0.42 | 0.1 | 13.7 | C1 |
| 28 | 15181 | CATAATGT | ATGnATG | 52.55 | 1.21 | 1.72 | 0.08 | 0.1 | 3.56 | C1 |
| 29 | 14980 | GAATATGG | ATGnATG | 47.21 | 2.85 | 1.67 | 0.45 | 0.5 | 2.1 | C1 |
| 30 | 14785 | TAAAATGC | ATGnATG | 44.7 | 2.25 | 1.65 | 0.35 | 0.3 | 2.79 | C1 |
| 31 | 15208 | CGCTATGT | ATGnATG | 43.91 | 1.37 | 1.64 | 0.14 | 0 | 32.29 | C1 |
| 32 | 14990 | GATCATGG | ATGnATG | 43.82 | 3.3 | 1.64 | 0.52 | 0.3 | 3.61 | C1 |
| 33 | 30919 | TACGCTGA | CTGnATG | 41.98 | 1.77 | 1.62 | 0.25 | 0.5 | 1.17 | C1 |
| 34 | 14341 | AACAATGA | ATGnATG | 40.98 | 1.32 | 1.61 | 0.12 | 0.2 | 1.88 | C1 |
| 35 | 63689 | TAGATTGA | TTGnATG | 40.62 | 2.72 | 1.61 | 0.43 | 0.3 | 2.67 | C1 |

| | | | | | | | | | | |
|---|---|---|---|---|---|---|---|---|---|---|
| 36 | 11140 | GAATAGGT | other | 39.74 | 1.11 | 1.6 | 0.05 | 0.3 | 1.14 | C1 |
| 37 | 14599 | AACGATGC | ATGnATG | 39.5 | 4.59 | 1.6 | 0.66 | 0.7 | 2.28 | C1 |
| 38 | 14977 | GAAAATGG | ATGnATG | 38.2 | 1.18 | 1.58 | 0.07 | 0.2 | 2.22 | C1 |
| 39 | 14852 | AAATATGG | ATGnATG | 38.08 | 1.55 | 1.58 | 0.19 | 0.1 | 7.31 | C1 |
| 40 | 14891 | AGGGATGG | ATGnATG | 37.66 | 1.3 | 1.58 | 0.11 | 0.3 | 1.57 | C1 |
| 41 | 14533 | TACAATGA | ATGnATG | 36.48 | 1 | 1.56 | 0 | 0.3 | 1.04 | C1 |
| 42 | 15234 | GAACATGT | ATGnATG | 35.49 | 3.02 | 1.55 | 0.48 | 0.7 | 1.4 | C1 |
| 43 | 15061 | TCCAATGG | ATGnATG | 35.08 | 1.79 | 1.55 | 0.25 | 0.4 | 1.48 | C1 |
| 44 | 14893 | AGTAATGG | ATGnATG | 34.75 | 1.28 | 1.54 | 0.11 | 0.4 | 1.21 | C1 |
| 45 | 56423 | CGCGTCTA | other | 33.78 | 1.46 | 1.53 | 0.16 | 0.2 | 3.13 | C1 |
| 46 | 14693 | CGCAATGC | ATGnATG | 33.6 | 1.01 | 1.53 | 0 | 0.1 | 5.95 | C1 |
| 47 | 48037 | GGCAGTGT | GTGnATG | 33.13 | 1.81 | 1.52 | 0.26 | 0.2 | 2.58 | C1 |
| 48 | 14877 | ACTAATGG | ATGnATG | 33 | 1.66 | 1.52 | 0.22 | 0.2 | 3.26 | C1 |
| 49 | 14664 | CACTATGC | ATGnATG | 31.45 | 1.72 | 1.5 | 0.24 | 0.5 | 1.1 | C1 |
| 50 | 2223 | GGTGAAGA | other | 30.95 | 2.69 | 1.49 | 0.43 | 0.4 | 2.39 | C1 |
| 51 | 14607 | AATGATGC | ATGnATG | 30.72 | 2.51 | 1.49 | 0.4 | 0.2 | 4.23 | C1 |
| 52 | 15302 | TACCATGT | ATGnATG | 30.56 | 1.17 | 1.49 | 0.07 | 0.4 | 1.06 | C1 |
| 53 | 63596 | CGGTTTGA | TTGnATG | 30.46 | 2.27 | 1.48 | 0.36 | 0.5 | 1.42 | C1 |
| 54 | 15109 | AACAATGT | ATGnATG | 30.37 | 1.19 | 1.48 | 0.08 | 0.1 | 2.8 | C1 |
| 55 | 55762 | TCACTCGC | other | 28.54 | 1.01 | 1.46 | 0 | 0.2 | 1.9 | C1 |
| 56 | 48119 | TTCGGTGT | GTGnATG | 28.17 | 1.15 | 1.45 | 0.06 | 0.2 | 1.74 | C1 |
| 57 | 62020 | CAATTTAG | other | 28.11 | 1.29 | 1.45 | 0.11 | 0.3 | 1.55 | C1 |
| 58 | 14437 | CGCAATGA | ATGnATG | 27.87 | 1.68 | 1.45 | 0.23 | 0 | 19.8 | C1 |
| 59 | 14669 | CATAATGC | ATGnATG | 26.91 | 1.6 | 1.43 | 0.2 | 0.1 | 4.19 | C1 |
| 60 | 57578 | TGGCTGAA | other | 26.51 | 2.5 | 1.42 | 0.4 | 0.7 | 1.26 | C1 |
| 61 | 14402 | CAACATGA | ATGnATG | 26.45 | 3.38 | 1.42 | 0.53 | 0.8 | 1.42 | C1 |
| 62 | 20992 | TTTTCCAC | other | 26.24 | 2.73 | 1.42 | 0.44 | 0.6 | 1.41 | C1 |
| 63 | 37557 | GTCAGCAG | other | 26.05 | 1.23 | 1.42 | 0.09 | 0.4 | 1.01 | C1 |
| 64 | 14921 | CAGAATGG | ATGnATG | 25.65 | 3.99 | 1.41 | 0.6 | 1 | 1.31 | C1 |
| 65 | 29701 | AACACTCA | other | 25.32 | 7.78 | 1.4 | 0.89 | 0.2 | 15.94 | C1 |
| 66 | 15241 | GAGAATGT | ATGnATG | 25.16 | 1.35 | 1.4 | 0.13 | 0.1 | 7.95 | C1 |
| 67 | 14413 | CATAATGA | ATGnATG | 24.83 | 2.87 | 1.39 | 0.46 | 0.6 | 1.73 | C1 |
| 68 | 65290 | AAGCTTTT | other | 24.34 | 1.96 | 1.39 | 0.29 | 0.5 | 1.3 | C1 |
| 70 | 14691 | CGAGATGC | ATGnATG | 24.03 | 1.51 | 1.38 | 0.18 | 0.5 | 1.08 | C1 |
| 69 | 14790 | TACCATGC | ATGnATG | 24.03 | 1.61 | 1.38 | 0.21 | 0.1 | 6.88 | C1 |
| 71 | 15245 | GATAATGT | ATGnATG | 23.24 | 1.69 | 1.37 | 0.23 | 0.5 | 1.15 | C1 |
| 72 | 48784 | GATTGTTG | other | 21.87 | 1.75 | 1.34 | 0.24 | 0.3 | 2.22 | C1 |

| | | | | | | | | | | |
|---|---|---|---|---|---|---|---|---|---|---|
| 73 | 32571 | ATGGCTTT | other | 19.7 | 1.37 | 1.29 | 0.14 | 0 | 21.45 | C1 |
| 74 | 14605 | AATAATGC | ATGnATG | 19.55 | 1.06 | 1.29 | 0.03 | 0.3 | 1.19 | C1 |
| 75 | 14350 | AATCATGA | ATGnATG | 18.8 | 1.98 | 1.27 | 0.3 | 0.4 | 1.67 | C1 |
| 76 | 48154 | ACGCGTTA | other | 18.29 | 1.15 | 1.26 | 0.06 | 0.2 | 2.09 | C1 |
| 77 | 14991 | GATGATGG | ATGnATG | 18.07 | 1.04 | 1.26 | 0.02 | 0.2 | 2.23 | C1 |
| 78 | 47885 | AATAGTGT | GTGnATG | 16.73 | 1.09 | 1.22 | 0.04 | 0.1 | 2.44 | C1 |
| 79 | 9205 | TTCAAGAT | other | 15.6 | 1.3 | 1.19 | 0.11 | 0.2 | 1.8 | C1 |
| 80 | 64422 | GGCCTTGT | TTGnATG | 14.21 | 1.32 | 1.15 | 0.12 | 0.3 | 1.48 | C1 |
| 81 | 41632 | GCTTGGAG | other | 14.16 | 3.08 | 1.15 | 0.49 | 0.5 | 1.91 | C1 |
| 82 | 4368 | AATTACAC | other | 13.57 | 2.12 | 1.13 | 0.33 | 0.3 | 2.22 | C1 |
| 83 | 36917 | ATCAGCAA | other | 13.09 | 1.31 | 1.12 | 0.12 | 0.1 | 5.59 | C1 |
| 84 | 48008 | GACTGTGT | GTGnATG | 12.89 | 2.5 | 1.11 | 0.4 | 0.4 | 2.36 | C1 |
| 85 | 15151 | AGTGATGT | ATGnATG | 12.47 | 2.27 | 1.1 | 0.36 | 0.1 | 7.64 | C1 |
| 86 | 40775 | CACGGCTT | other | 12.34 | 1.77 | 1.09 | 0.25 | 0.4 | 1.39 | C1 |
| 87 | 15056 | TATTATGG | ATGnATG | 12.13 | 1.14 | 1.08 | 0.06 | 0.2 | 1.62 | C1 |
| 88 | 52444 | TCGTTATA | other | 12.02 | 1.81 | 1.08 | 0.26 | 0 | 14.22 | C1 |
| 89 | 15633 | ACAAATTC | other | 11.55 | 15.92 | 1.06 | 1.2 | 0.3 | 20.85 | C1 |
| 90 | 47473 | CTAAGTGC | GTGnATG | 11.2 | 4.12 | 1.05 | 0.61 | 1.2 | 1.15 | C1 |
| 91 | 15081 | TGGAATGG | ATGnATG | 11.19 | 2.54 | 1.05 | 0.4 | 0.4 | 2.1 | C1 |
| 92 | 47909 | AGCAGTGT | GTGnATG | 11.14 | 1.5 | 1.05 | 0.18 | 0.1 | 3.54 | C1 |
| 93 | 34374 | CACCGACG | other | 10.14 | 1.43 | 1.01 | 0.16 | 0.2 | 2.11 | C1 |
| 94 | 15146 | AGGCATGT | ATGnATG | 10.13 | 2.19 | 1.01 | 0.34 | 0.6 | 1.23 | C1 |
| 95 | 48047 | GGTGGTGT | GTGnATG | 9.95 | 4.61 | 1 | 0.66 | 1.5 | 1.04 | C1 |
| 96 | 14896 | AGTTATGG | ATGnATG | 9.94 | 1.93 | 1 | 0.29 | 0.2 | 2.68 | C1 |
| 97 | 47919 | AGTGGTGT | GTGnATG | 9.88 | 1.01 | 0.99 | 0 | 0.1 | 3.97 | C1 |
| 98 | 47982 | CGTCGTGT | GTGnATG | 9.42 | 1.08 | 0.97 | 0.03 | 0.3 | 1.27 | C1 |
| 99 | 30450 | TTACCTCG | other | 9.28 | 2.49 | 0.97 | 0.4 | 0.4 | 2.3 | C1 |
| 100 | 47463 | CGCGGTGC | GTGnATG | 9.19 | 1.17 | 0.96 | 0.07 | 0.1 | 5.52 | C1 |
| 101 | 14755 | GGAGATGC | ATGnATG | 8.98 | 1.72 | 0.95 | 0.24 | 0.3 | 2.25 | C1 |
| 102 | 64517 | AACATTTA | other | 8.82 | 2.44 | 0.95 | 0.39 | 0.7 | 1.17 | C1 |
| 103 | 63815 | CACGTTGC | TTGnATG | 8.14 | 1.49 | 0.91 | 0.17 | 0.2 | 2.59 | C1 |
| 104 | 46584 | TTCTGTCC | other | 7.64 | 1.33 | 0.88 | 0.12 | 0.3 | 1.33 | C1 |
| 105 | 56566 | TTCCTCTA | other | 7.63 | 8.76 | 0.88 | 0.94 | 2.2 | 1.32 | C1 |
| 106 | 45556 | TTATGTAC | other | 7.55 | 2.82 | 0.88 | 0.45 | 0.9 | 1.1 | C1 |
| 107 | 47984 | CGTTGTGT | GTGnATG | 7.12 | 1.3 | 0.85 | 0.11 | 0.3 | 1.3 | C1 |
| 108 | 47957 | CCCAGTGT | GTGnATG | 6.16 | 4.31 | 0.79 | 0.63 | 0.9 | 1.52 | C1 |
| 109 | 11883 | CGGGAGTG | other | 5.59 | 1.63 | 0.75 | 0.21 | 0.2 | 2.56 | C1 |
